# Favorable outcome of pheochromocytoma in a dog with atypical Cushing’s syndrome and diabetes mellitus following medical treatment: a case report

**DOI:** 10.1186/s12917-019-2225-x

**Published:** 2020-01-03

**Authors:** Ga-Won Lee, Cho-Rong Yoo, Dan Lee, Hee-Myung Park

**Affiliations:** Department of Veterinary Internal Medicine, College of Veterinary Medicine, Konkuk University, # 1 Hwayang-dong, Gwang-jin-gu, Seoul, 143-701 South Korea

**Keywords:** Adrenal tumor, Atypical Cushing’s syndrome, Dog, Diabetes mellitus, Pheochromocytoma

## Abstract

**Background:**

Pheochromocytoma (PCC) has poor prognosis and adrenalectomy is hard to be performed, in case of caudal vena cava invasion. The long-term administration of phenoxybenzamine in PCC has not been reported in dogs.

**Case presentation:**

A 14-year-old castrated male Poodle dog presented with an abdominal mass. On physical examination, hypertension, increased lens opacity, calcinosis cutis, generalized alopecia, and systolic murmur were observed. Serum chemistry and urinalysis profiles revealed hyperglycemia, hypercholesterolemia, elevated liver enzymes, and glucosuria. Abdominal ultrasonography showed a right adrenal mass with invasion of the caudal vena cava, which was cytologically diagnosed as suspected PCC. An adrenal mass (width × height × length, 28 × 26 × 48 mm^3^) was found on computed tomography and diagnosed as PCC with increased plasma metanephrines and normetanephrines. An adrenocorticotropin hormone stimulation test showed elevated adrenal hormones (androstenedione, estradiol, progesterone, and 17-OH progesterone) with normal cortisol, compatible with atypical Cushing’s syndrome. The dog was managed with trilostane, phenoxybenzamine, and insulin therapy. Glycosylated hemoglobin and fructosamine levels gradually decreased, and hypertension resolved. In the 10-month follow-up period, the liver enzymes levels gradually decreased, and the clinical signs of the dog were well-controlled without deterioration.

**Conclusions:**

This case report describes long-term medical management without adrenalectomy of PCC complicated with atypical Cushing’s syndrome and DM.

## Background

Adrenal tumors are common in dogs, functionally active, and may secrete an excessive amount of one or more type of hormones, causing tumor-related syndromes [1]. Among them, pheochromocytoma (PCC) is a rare tumor, which is derived from chromaffin cells in the adrenal medulla [2–6]. PCC typically affects middle-aged-to-old dogs with over 50% of cases involving local invasion to the caudal vena cava (CVC) and surrounding soft tissues [2, 3, 7]. Metastases to the local lymph nodes, lungs, and liver have been reported in dogs [4, 5]. The clinical signs of PCC are typically nonspecific but can be acute and life threatening [2, 3, 5], including lethargy, tachyarrhythmias, hypertension, polyuria/polydipsia (PU/PD), and collapse [4, 7]. Excessive secretion of catecholamines from tumor tissues manifests these clinical features [2, 8]. The definitive diagnosis of PCC relies on histopathology of the adrenal mass [3, 7], but plasma free metanephrine (fMN) and normetanephrine (fNMN) concentrations can be useful to identify PCC in both humans [7] and dogs [3, 9]. Moreover, cytology of the primary adrenal tumor is helpful in distinguishing the cortical tumors from the medullary ones [1]. In general, PCC is more aggressive in dogs than in humans [2].

This is a case report that describes the clinical manifestations and favorable outcome following intensive medical treatment of PCC in a dog complicated with atypical Cushing’s syndrome and diabetes mellitus (DM).

## Case presentation

A 14-year-old castrated male Poodle dog was referred for evaluation of an abdominal mass. The dog had a history of PU/PD and hypertension and was diagnosed with DM 6 months before. Irbesartan, an anti-hypertensive agent, and intermediate acting insulin for controlling DM were administered before the visit.

**Fig. 1:**
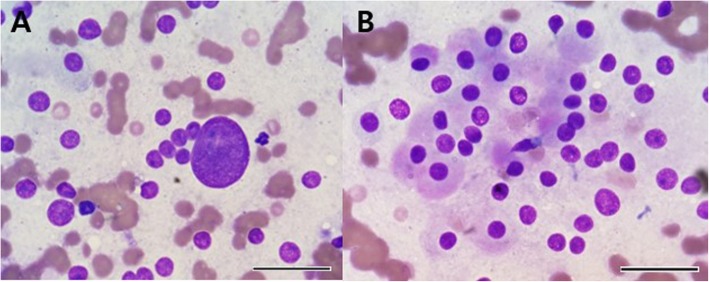
Cytology from the fine-needle aspiration biopsy of a right adrenal mass diagnosed as pheochromocytoma in a dog. Neuroendocrine cells, with naked nuclei, anisokaryosis, prominent nucleoli, and coarse chromatin are seen (**a**). Polygonal cells containing moderate amounts of slightly basophilic granular cytoplasm are predominant (**b**). Diff-Quick stain; Bar = 25 μm (**a** & **b**)

Physical examination revealed hypertension (systolic/diastolic blood pressure [BP], 155/108), increased lens opacity, calcinosis cutis, generalized alopecia, and systolic murmur (grade 3). Hematologic and serum biochemical profiles showed hyperglycemia, elevated liver enzymes, and hypercholesterolemia (Table 1). DM was poorly controlled, with a glycosylated hemoglobin (HbA1c) of 8.4% (68 mmol/mol; reference interval, 0.6–2.7%) [10]. Urinalysis showed glucosuria (4+, 1000 mg/dL). Radiograph showed cardiomegaly (vertebral heart score, 11.5v), a mild bronchointerstitial pattern on the overall lung field, and hepatomegaly. Abdominal ultrasonography showed a right adrenal mass with invasion of CVC and increased hepatic parenchymal echogenicity with gall bladder sludge. Differential diagnosis for the adrenal mass included adrenal-dependent hyperadrenocorticism, hyperaldosteronism, and PCC. Fine-needle aspiration biopsy of the adrenal mass showed predominant naked nuclei, suspected as neuroendocrine cells, and polygonal cells containing a moderate amount of slightly basophilic and finely granular cytoplasm, which originated from the adrenal medulla (Fig. 1). There were a few clusters of adrenal cortical cells with cytoplasmic vacuolation.

**Table 1:** Complete blood count and serum biochemical results of a dog with pheochromocytoma, atypical Cushing’s syndrome, and diabetes mellitus

Parameters	D0	D19	D39	D61	D90	D125	Reference interval
WBC (10^9^/L)	9.04	9.03	8.39	10.91	10.87	8.85	5.05–16.7
HCT (%)	50.6	51.5	44.6	46.1	40.6	41.3	37.3–61.7
PLT (10^3^/μL)	452	409	450	473	473	399	148–484
ALT (U/dL)	232	842	261	189	232	175	10–100
AST (U/dL)	83	710	66	177	84	52	0–50
ALP (U/dL)	1019	632	628	497	619	315	23–212
GGT (U/dL)	21	17	15	11	13	14	100–200
Glucose^a^ (mg/dL)	234	316	341	246	351	220	70–143
TChol (mg/dL)	434	392	–	–	–	266	110–320
Fructosamine (μmol/L)	465	–	534	504	343	314	177–314
HbA1c (%)	8.4	–	9.2	–	–	6.6	0.6–2.7^16^

*D* days after first examination, *WBC* white blood cells, *HCT* hematocrit, *PLT* platelet, *ALT* alanine transaminase, *AST* aspartate transaminase, *ALP* alkaline phosphatase, *GGT* gamma-glutamyl transferase, *TChol* total cholesterol; ^a^nadir of the glucose levels

**Fig. 2:**
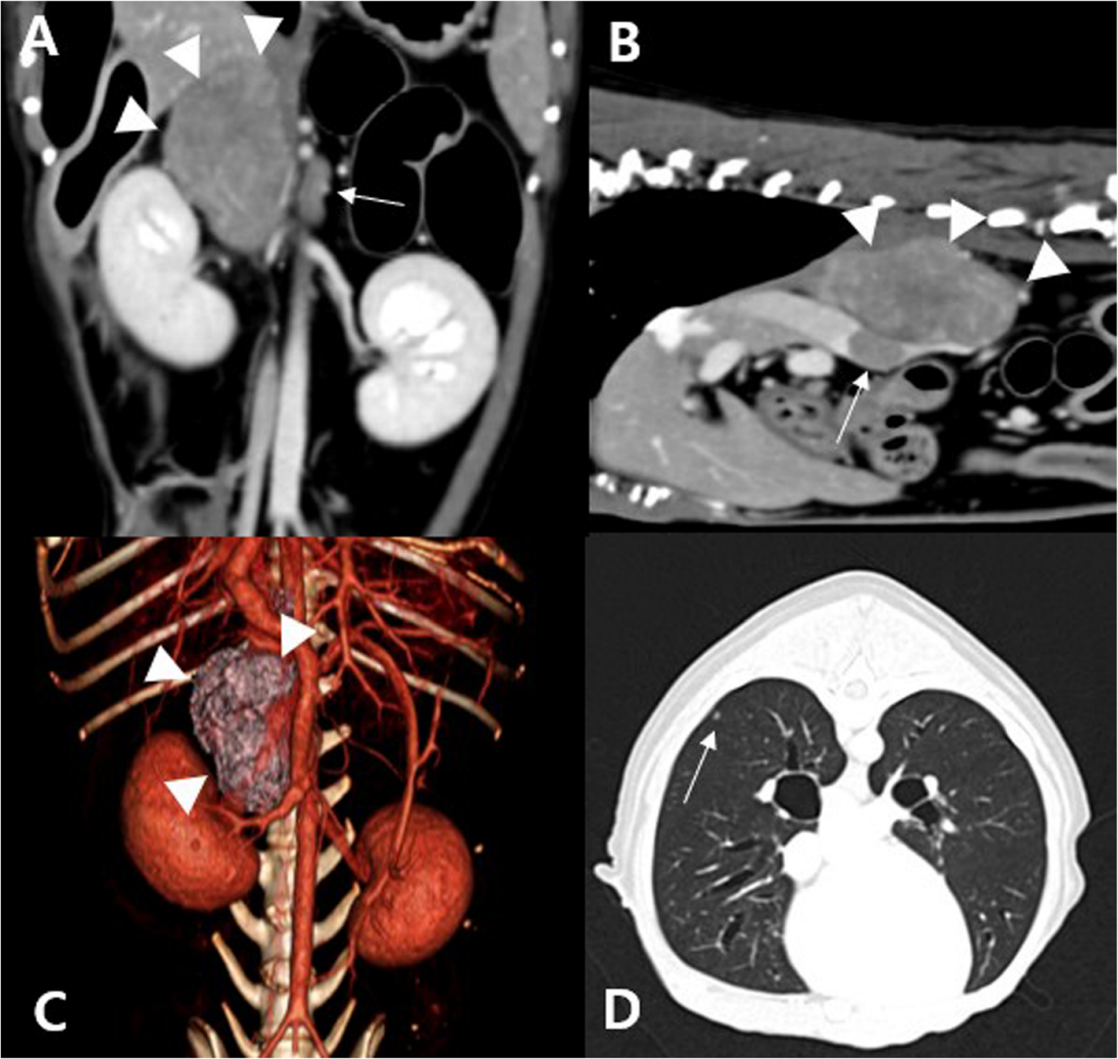
Computed tomography (CT) showing dorsal (**a**), sagittal (**b**), and transverse (**d**) images and a dorsal view of the 3D volume reconstructed renderings created from the CT images (**c**) of a dog diagnosed with pheochromocytoma. Heterogenous attenuation and multiple mineralization of the mass (arrow heads) are observed (**a** & **b**). The size of the right adrenal mass (arrow heads) is width × height × length = 28 × 26 × 48 mm^3^ (**a** & **c**) and that of the left adrenal gland (an arrow) is width × height × length = 5.6 × 2.3 × 14 mm^3^ (**a**). Prominent caudal vena cava invasion (an arrow) is also revealed (**b**). A small nodule (an arrow) in the right caudal lobe is observed (**d**)

Cytologic evaluation of the mass suggested that it could be originated from the adrenal medulla. Computed tomography (CT) was performed to confirm the origin of the mass, evaluate local or distant metastasis, and prepare the therapeutic plan. CT revealed an enlarged, mineralized right adrenal mass (width × height × length, 28 × 26 × 48 mm^3^) with CVC invasion and a nodule in the right lung lobe, indicating suspected distant metastasis from the malignant adrenal tumor (Fig. 2). An adrenocorticotropin hormone (ACTH; Synacthen; Dalim Bio Tech, Korea) stimulation test was performed to identify the presence of adrenal-dependent hyperadrenocorticism (University of Tennessee, Knoxville, TN, USA). The adrenal hormone panel showed elevated adrenal hormones (androstenedione [pre-stimulation, 4.53 ng/mL; reference interval, 0.05–0.36 ng/mL; post-stimulation, 6.18 ng/mL; reference interval, 0.24–2.90 ng/mL], estradiol [pre-stimulation, 87.7 pg/mL; reference interval, 23.1–65.1 pg/mL; post-stimulation, 72.4 pg/mL; reference interval, 23.3–69.4 pg/mL], progesterone [pre-stimulation, < 0.20 ng/mL; reference interval, < 0.20 ng/mL; post-stimulation, 2.44 ng/mL; reference interval, 0.22–1.45 ng/mL], and 17-OH progesterone [pre-stimulation, 1.40 ng/mL; reference interval, 0.08–0.22 ng/mL; post-stimulation, 11.14 ng/mL; reference interval, 0.25–2.63 ng/mL]) with normal cortisol (pre-stimulation, 3.4 μg/dL; reference interval, < 1.0–5.6 μg/dL; post-stimulation, 9.1 μg/dL; reference interval, 7.1–15.1 μg/dL) in both pre- and post- ACTH stimulation tests (Table 2). Table 3 shows the results of plasma fMN and fNMN, which were measured to investigate adrenal medullary involvement. The findings were consistent with PCC in dogs (fMN, > 4.18 nmol/L; fNMN, > 5.52 nmol/L) [3]. Based on the laboratory and clinical examinations, DM concurrent with PCC and atypical Cushing’s syndrome were suspected. Additionally, an echocardiograph revealed mitral valve degeneration and regurgitation, indicating myxomatous mitral valve degeneration (MMVD).

**Table 2:** Adrenal hormone concentrations before and after adrenocorticotropin hormone stimulation in a dog with atypical Cushing’s syndrome

Parameters	Baseline	Reference interval	After stimulation	Reference interval
Cortisol (μg/dL)	3.4	< 1.0–5.6	9.1	7.1–15.1
Androstenedione (ng/mL)	4.53	0.05–0.36	6.18	0.24–2.90
Estradiol (pg/mL)	87.7	23.1–65.1	72.4	23.3–69.4
Progesterone (ng/mL)	< 0.2	< 0.20	2.44	0.22–1.45
17-OH Progesterone (ng/mL)	1.4	0.08–0.22	11.14	0.25–2.63
Testosterone (ng/dL)	< 15.0	< 15.0–24.0	< 15.0	< 15.0–42.0
Aldosterone (pg/mL)	23.8	6.7–253.6	99.4	55.6–737.2

**Table 3:** Plasma free metanephrine and free normetanephrine levels in a dog with pheochromocytoma

Parameters	D0	D39	D90	Reference interval
fMN	27.36	22.90	19.40	0.68–3.08^3^
fNMN	94.28	109.28	103.20	1.59–4.17^3^

*D* days after first examination, *fMN* free metanephrine, *fNMN* free normetanephrine

Furosemide (1 mg/kg orally q12h; Handok, Korea) and trilostane (1 mg/kg orally q12h; Dechra, UK) were initiated to control MMVD and atypical Cushing’s syndrome. Clopidogrel (3 mg/kg orally q24h; Sinil, Korea) was additionally prescribed as an anti-coagulant. Insulin therapy (isophane insulin; 0.35 IU/kg; Humulin, Lilly, USA.) and irbesartan (5 mg/kg orally q24h; Sanofi Winthrop Industrie, France) were continued to control DM and hypertension.

On day 19, the liver enzyme levels abruptly increased, and trilostane dosage was increased to twice that of the initial dosage. Moreover, irbesartan was switched to hydralazine (0.5 mg/kg orally q12h; Samjin, Korea), considering the risk of hyperkalemia. On day 25, systolic BP was 163 mmHg, and glucose maximum and nadir were 328 mg/dL and 242 mg/dL, respectively. Therefore, the hydralazine dosage was increased to 1 mg/kg, orally, q12h, and isophane insulin was switched to caninsulin (1 unit twice a day; MSD, Korea). Until day 90, the caninsulin dosage was 1.8 units, but the glycemic curve was not well-controlled, and the fructosamine level was over 343 μmol/L, indicating poor response to the medical treatment. Therefore, phenoxybenzamine (PBZ; 0.25 mg/kg orally q12h; Aristo, German) was added to control hypertension and improve glucose intolerance. One week later, systolic BP decreased to 131 mmHg, and glucose maximum and nadir also decreased to 265 mg/dL and 169 mg/dL, respectively. One month after the start of PBZ, fructosamine decreased to 314 μmol/L, and HbA1c decreased to 6.6% (49 mmol/mol), showing a favorable outcome (Table 1, day 125). The treatment was continued for 10 months, and the liver enzyme levels gradually decreased, with well-controlled DM and hypertension. Moreover, the owner perceived increased activity of the dog, and the general condition of the dog improved with no other side effects.

## Discussion and conclusions

DM is a common complication of PCC in humans, resulting from impaired glucose tolerance due to catecholamine excess [11]. Glucose intolerance or DM can occur in 35–50% of patients with PCC, as increased catecholamine levels induce downregulation of insulin secretion and upregulation of insulin resistance [12]. Moreover, glucose uptake decreases, and gluconeogenesis and glycogenolysis increase as sequelae of excessive catecholamine levels [11, 12]. Hyperglycemia changes to normoglycemia after resection of PCC in 79% of patients with PCC and DM [11]. In humans, prevalence of DM concurrent with PCC correlates to large and symptomatic tumors [11], but until recently, there was no information about the risk factors of DM in dogs with PCC.

PBZ is an α-adrenergic antagonist, which irreversibly and noncompetitively binds to both α-1 and α-2 adrenergic receptors, thereby blocking the α-adrenergic effect to the circulating epinephrine and norepinephrine [5, 7]. In humans with PCC, most patients receive PBZ for days to weeks before adrenalectomy to control BP in the perioperative period [13], which also decreases perioperative mortality in dogs with PCC [7]. In cases of non-resectable PCC, medical treatment with PBZ is indicated to manage hypertension [5, 14]. The adverse effects of PBZ include nasal stuffiness and postural hypotension in humans [13], and hypotension, miosis, and tachycardia in dogs [15]. Although the accurate dose, frequency, and duration of PBZ administration to adequately achieve the desired effects have not been defined for dogs [7], the dog in this case showed a favorable outcome in 10 months after start of low-dose PBZ.

Hypertension is a serious sign of PCC and the principal cause of death from a tumor in humans [13]. In this case, the possible causes of hypertension included DM, atypical Cushing’s syndrome, and PCC. To manage the dog’s hypertension, trilostane and hydralazine were administrated. However, there was no response to the treatment; therefore, PBZ was administered additionally. In humans, blocking the α-adrenergic receptors can not only control hypertension but also improve glucose intolerance and insulin release [12, 14]. In this case, after PBZ administration, the dog’s glycemic curve was well-controlled, and HbA1c had remarkably improved. Moreover, hypertension was resolved. Considering the history of poorly controlled DM and a clinically favorable response to PBZ, PCC could have led to glucose intolerance, which progressed to DM. Similarly, if glycemic control is difficult in dogs with DM, other possible causes, including insulin resistance, should be considered, such as PCC, hyperadrenocorticism, and obesity [16].

In this case, the definitive diagnosis of PCC could not be made without histologic examination of the dog’s adrenal gland. However, the increased plasma fMN and fNMN levels, normal-sized left adrenal gland, clinical presentation, and cytologic findings led to the presumptive diagnosis of PCC. Moreover, a complete adrenal panel was helpful in diagnosing atypical Cushing’s syndrome. Increased adrenal sex hormone concentrations have been reported in dogs with non-cortisol-secreting adrenocortical tumors [17]; thus, non-cortisol-secreting adrenocortical tumor could be concurrent with PCC, inducing atypical Cushing’s syndrome in this case. However, ectopic ACTH secretion from PCC could have occurred, triggering atypical Cushing’s syndrome by up-regulation of ACTH secretion. Although the etiopathogenesis of atypical Cushing’s syndrome is unknown, this report describes a rare case of combined PCC and atypical Cushing’s syndrome in a dog.

PCC has poor prognosis in both humans and dogs [2, 14], and the definitive treatment of PCC requires adrenalectomy [4]. However, in this case, adrenalectomy could not be performed because of CVC invasion, and the dog had poor prognostic factors, such as a large-sized tumor and suspected pulmonary metastasis [7]. However, in the 10-month follow-up period, the clinical signs gradually improved, and there were no side effects from the administered drugs, thereby increasing the quality of life.

In conclusion, plasma fMN and fNMN levels could aid in the diagnosis of PCC, allowing appropriate and rapid targeted therapy in cases of an adrenal mass by differentiating between an adrenal cortex tumor and PCC. Although the dog had severe multiple endocrine diseases including PCC, atypical Cushing’s disease and DM, the diseases were managed successfully with medical therapy and without surgery. In particular, PBZ lead to clinical improvement in hypertension and glycemic control in the dog. This is a case report that describes the clinical manifestations and favorable outcome following intensive medical treatment of PCC in a dog with atypical Cushing’s syndrome and DM.

## Data Availability

All the data are presented in the main paper and accompanying figures.

## Abbreviations

ACTH: Adrenocorticotropin hormone
BP: Blood pressure
CT: Computed tomography
CVC: Caudal vena cava
DM: Diabetes mellitus
fMN: Free metanephrine
fNMN: Free normetanephrine
HbA1c: Glycosylated hemoglobin
MMVD: Mitral valve degenerative disease
PBZ: Phenoxybenzamine
PCC: Pheochromocytoma
PU/PD: Polyuria/polydipsia
